# Impact of Cardiopulmonary Rehabilitation on Patients with Heart Failure Reduced Ejection Fraction and Preserved Ejection Fraction

**DOI:** 10.3390/jcm14113815

**Published:** 2025-05-29

**Authors:** Sabine Gempel, Jenna Kologie, Taylor Wright, Destini Decinti, Lawrence Cahalin

**Affiliations:** 1 Department of Physical Therapy, Miller School of Medicine, University of Miami, Coral Gables, FL 33146, USA; jmk302@miami.edu (J.K.); tlw80@miami.edu (T.W.); dxd1165@miami.edu (D.D.); l.cahalin@miami.edu (L.C.); 2 Department of Cardiac Rehabilitation, University of Miami Hospital, Miami, FL 33136, USA

**Keywords:** cardiopulmonary rehabilitation, cardiac rehabilitation, heart failure with reduced ejection fraction, heart failure with preserved ejection fraction, heart failure, exercise training

## Abstract

**Background/Objectives**: The prevalence of heart failure with preserved ejection fraction (HFpEF) is expected to surpass that of heart failure with reduced ejection fraction (HFrEF), yet it remains under-researched. Compared to HFrEF, patients with HFpEF have similarly poor survival rates, physical impairments, and quality of life (QOL) and similar improvements following exercise training. However, Medicare currently excludes coverage for cardiopulmonary rehabilitation (CR) for HFpEF. The purpose of this study was to evaluate the impact of HF at baseline and the effects of CR in both subtypes. **Methods**: Ninety-nine patients (forty-three with HFrEF and fifty-six with HFpEF) who completed CR were included. Demographic data and outcome measures were assessed pre- and post-CR, including weight, body mass index (BMI), 5x-sit-to-stand (5xStS), timed-up-and-go (TUG), 6-minute walk test (6MWT), Ferrans and Powers Quality of Life (F&P QOL), waist circumference, BERG balance, and Patient Health Questionnaire-9 (PHQ-9). Independent and paired t-tests were performed with statistical significance set at *p* < 0.05. **Results**: At baseline, compared to patients with HFrEF, patients with HFpEF were older with a significantly lower 6MWT distance (350.6 m vs. 299.6 m), lower BERG balance scores (52/56 vs. 49/56, respectively), and a 5xSTS score indicating a fall risk (16.9 ± 6.5). Following CR, both groups had significant improvements in all functional and self-reported outcome measures (*p* < 0.001), with no significant differences between HF subtypes. Patients with HFpEF also had a significant improvement in waist circumference. **Conclusions**: Compared to patients with HFrEF, patients with HFpEF presented with similar or greater impairments and had similar or greater improvements following CR. These results underscore the effectiveness of CR for HFpEF management and the need for insurance coverage.

## 1. Introduction

The overall incidence of heart failure (HF) in the United States seems to be stabilizing or even decreasing. However, the incidence of HF with preserved ejection fraction (HFpEF) continues to increase, and the prevalence is expected to surpass HF with reduced ejection fraction (HFrEF) in the near future [1]. While our understanding of the pathophysiology and diagnosis of HFpEF have evolved and improved, it remains under-recognized and under-researched and there are limited therapeutic options [1].

Compared to HFrEF, patients with HFpEF have similarly poor survival rates with 5-year all-cause mortality rates of 75.3% and 75.7%, respectively [1], as well as a high burden of sarcopenia and frailty, reduced quality of life (QOL) [2], and impaired peak oxygen consumption (VO2 peak) [3]. Physical inactivity and decreased aerobic capacity are associated with an increased risk of developing HFpEF and, subsequently, a worsening QOL and mortality risk [1,3]. While conventional therapy has been largely ineffective at addressing these outcomes, exercise has been shown to be an effective adjunct therapy that directly addresses the primary symptom that is associated with HFpEF, namely exercise intolerance [2]. Medicare initially only covered cardiopulmonary rehabilitation (CR) for patients with coronary artery disease; however, this coverage expanded to include HFrEF [4] in 2014 and peripheral arterial disease in 2017 [5] as the evidence supporting supervised exercise in a multidisciplinary environment grew. Yet, Medicare does not currently cover HFpEF, despite the 2023 scientific statement from the American Heart Association (AHA) and American College of Cardiology (ACC) supporting the inclusion of and insurance coverage for HFpEF [2], as well as the AHA’s Level 1A recommendation for a CR referral based on evidence that exercise training leads to improved exercise capacity, QOL, hospital readmission rates, cardiac function, and potentially mortality rates [1,3]. Additionally, the data suggest that patients with HFpEF have a similar, if not greater, improvement following an exercise intervention [2].

The majority of research on HF focuses solely or predominantly on patients with HFrEF. Even when both groups are included, the authors rarely separate patients with HFpEF and HFrEF to compare baseline presentation and response to exercise. To the best of our knowledge, only one other study compares the impact of CR on physical function and QOL between these HF groups [6]. Therefore, the purpose of our study is to examine the impact of HFrEF and HFpEF on functional performance and emotional state as well as the changes seen following CR completion.

## 2. Materials and Methods

A retrospective chart review was performed for patients who attended CR with a HF diagnosis at the University of Miami Health (UMH) Systems between June 2018 and June 2023. This study was approved by the University of Miami’s Institutional Review Board (protocol #20230667) on 9 August 2023. Using the updated HF definitions, HFpEF was defined as having an ejection fraction (EF) of ≥50%, and HFrEF was defined as an EF ≤40% [7]. To be included in this study, subjects had to be 18 years and older who had attended and completed CR at UMH during the 5-year time period with either a referral diagnosis or co-morbid diagnosis of HFrEF, HFpEF, or cardiomyopathy. The patients’ most recent echocardiogram prior to starting CR was used to classify patients as having HFrEF or HFpEF. Since HFpEF is not a covered diagnosis for CR, these patients qualified for CR via another cardiac or pulmonary diagnosis. Patients’ insurance typically covers up to 36 sessions of CR; however, some patients are able to complete the program with fewer than 36 sessions if all of the goals are met. Our CR program includes a medical director who is a cardiologist, a registered nurse as well as a doctor of nurse practice, doctors of physical therapy who are board-certified cardiovascular and pulmonary specialists, and referrals to a registered dieticians, psychologists, and smoking cessation programs as needed. Our program creates an individualized plan of care, which includes aerobic exercise, strength training for peripheral and respiratory muscles, balance training, breathing techniques, and stretching, as indicated following the initial evaluation as well as considerations related to a patient’s goals, personal and employment roles, and responsibilities. Additionally, the program aims to achieve moderate to high intensities through a combination of rate of perceived exertion, percentage of max heartrate, vital sign response, and patient symptoms. Extensive education is also provided consistent with the American Association of Cardiovascular and Pulmonary Rehabilitation guidelines and patient-specific needs, including nutrition counseling, risk factor management, and physical activity counseling [8]. Individuals were excluded if they were <18 years old or had a severe musculoskeletal impairment preventing them from performing aerobic exercise and physical outcome measures.

Data were collected from the electronic medical record of each patient, including age, sex, height, weight, body mass index (BMI), number of medications, comorbidities, number of sessions completed, and baseline and post-CR assessments. These assessments were performed using standardized methods and included weight, body mass index (BMI), 5x-sit-to-stand (5xStS), timed-up-and-go (TUG), 6-minute walk test (6MWT), Ferrans & Powers Quality of Life (F&P QOL), waist circumference, BERG balance scale (BBS), and the Patient Health Questionnaire-9 (PHQ-9).

The 6MWT is a submaximal aerobic/functional capacity and endurance assessment, with a score <300 m associated with poorer survival and outcomes [9,10]. The TUG assesses fall risk, with a score of 13.5 s or greater indicating an increased risk of falls [11]. The 5xStS measures functional lower extremity strength and power, with a score >16 s indicating a fall risk [12]. The BBS examines static and dynamic balance activities of varying difficulty, where scores of 40 or less may indicate an increased risk of falling [13]. The PHQ-9 is a short self-reported screening tool used to assess depression severity, with scores of 0–4, 5–9, 10–14, 15–19, and 20–27 representing minimal, mild, moderate, moderately severe, and severe depression, respectively. The PHQ-9 has been shown to be a reliable and valid measure of depression severity [14]. The F&P QOL index produces 5 scores between 0 and 30, with higher scores reflecting a better QOL, and is recommended by the American Association of Cardiovascular and Pulmonary Rehabilitation for CR use [15].

Statistical analyses included assessment of the normality of data using the Kolmogorov–Smirnov and Shapiro–Wilk tests, calculation of descriptive statistics, and comparison of the demographic and clinical characteristics of patients with HFrEF versus HFpEF. Most variables were normally distributed; thus, for consistency, parametric statistics were used to compare between and within groups. All analyses were performed using IBM^®^ SPSS^®^ Statistics version 26 with the level of significance set at a *p*-value < 0.05.

## 3. Results

The demographic and clinical characteristics of the patients are shown in Table 1. A total of 99 patients (43 HFrEF and 56 HFpEF) were referred to and completed CR.

### 3.1. Baseline Characteristics of the Overall Population

At baseline, patients with HFpEF were older than patients with HFrEF (mean age ± SD was 68.9 ± 11.7 and 62.2 ± 13.7, respectively). There were no baseline differences in height, weight, BMI, waist circumference, number of medications, or number of comorbidities. In fact, the proportions of subjects with atrial fibrillation (10.5% vs. 12%), type 2 diabetes (37% vs. 38%), and hypertension (63% vs. 69%) were not significantly different between subjects with HFrEF and HFpEF, respectively. Both patients with HFpEF and HFrEF had an elevated mean waist circumference (103.9 cm and 103.1 cm, respectively). There were also no baseline differences in the TUG, PHQ9, or F&P QOL. However, patients with HFpEF walked significantly less during the 6MWT than patients with HFrEF (299.6 m vs. 350.6 m, respectively; *p* < 0.001), had a worse BBS than patients with HFrEF (49/56 vs. 52/56, respectively; *p* = 0.08), and had a 5xSTS time that was above the fall risk cutoff score of 16.

### 3.2. Outcomes Following Cardiac Rehabilitation

Following CR, there were no significant differences in any outcome measures, nor the number of CR sessions completed between patients with HFrEF and HFpEF. Both groups had statistically significant improvements in TUG, 6MWT, 5xStS, BBS, PHQ9, and F&P QOL. Patients with HFpEF also exceeded the 300 m threshold 6MWT distance and had a significant improvement in waist circumference (*p* < 0.001) and a near significant improvement in BMI (*p* = 0.05), while the HFrEF group had only a near significant improvement in waist circumference (*p* = 0.05) and did not have an improvement in BMI (Table 2).

## 4. Discussion

Patients with HF displayed functional and emotional impairments that improved significantly following CR. Compared to patients with HFrEF, those with HFpEF presented with similar or greater baseline impairments and demonstrated similar or greater improvements following CR, which is similar to the findings of Pandey et al. [6]. To the best of our knowledge, this is the second study to directly compare these HF subtypes before and after CR and the first to include this variety of physical and emotional outcome measures. Our results add valuable evidence to an underrepresented HF subgroup, suggesting that patients with HFpEF require and would benefit from CR. These results are important because CMS does not currently cover CR for patients with HF with an EF >35%, despite the growing evidence supporting this need.

### 4.1. Heart Failure Risk Factors and Impairments by HF SubType at Baseline

While both HFrEF and HFpEF share most of the traditional HF risk factors, the risk for HFpEF appears to be associated with obesity, insulin resistance, and physical inactivity more so than HFrEF [1]. At baseline, patients with HFpEF had a mean BMI classifying them as “obese”, whereas patients with HFrEF, on average, were “overweight”. Both HF subtypes had a mean waist circumference above the optimal cutoff values [16]. Patients with HFpEF also presented with significantly poorer 6MWT performance, suggesting decreased aerobic/functional capacity and a 5xStS score above the cutoff score, indicating increased risk of falls and decreased lower-extremity strength and power, which could potentially be explained in part by the significantly older age of the patients with HFpEF. Otherwise, all other outcome measures were similarly impacted and showed no significant difference between the two HF subtypes at baseline. This is supported by previous research showing that peak VO2 and 6MWT are both severely reduced by a similar magnitude in patients with HFpEF and HFrEF [2,3,4,5,6].

A common complication associated with the diagnosis of HF includes reduced QOL [1]. Depression occurs in approximately 55–60% of patients with HF and is associated with poorer QOL [17]. At baseline, our results showed the mean PHQ-9 scores for both patients with HFrEF and HFpEF were in the “minimal depression” category, with scores of 4.1 ± 4.5 and 4.8 ± 4.6 and an F&P QOL mean score of 25.0 ± 3.7 and 24.0 ± 4.0, respectively. Similarly, Nolte et al. found that patients with HFpEF in the intervention group and control group had baseline PHQ-9 scores of 7 ± 6 (“mild depression”) and 5 ± 5 (“minimal depression”), respectively [18]. Similarly, the HF-Action study on patients with HFrEF found a baseline Beck Depression Inventory-II median score of 8 (“minimal depression”) [19]. A study by Davidson et al. looking at both HF subtypes using the Minnesota Living with Heart Failure Questionnaire (MLHFQ) found a mean baseline score of 44 (“moderate QOL” [20]) and 52 (“poor QOL” [20]) for the intervention and control groups, respectively [21]. These studies suggest that patients with HFpEF and HFrEF are similarly impacted by their HF diagnosis, which supports our findings.

### 4.2. Impact of Exercise

The primary symptom associated with HF is exercise intolerance [1]. The pathophysiologic mechanisms of HFpEF include cardiac, endothelial, and skeletal muscle dysfunction, much of which can be improved through exercise [2]. We found that both patients with HFrEF and HFpEF had statistically significant improvements in functional performance measures, including TUG, 6MWT, 5xStS, and BBS, as well as self-reported outcomes regarding QOL and depression risk. Additionally, patients with HFpEF had significant improvements in waist circumference and 6MWT, which was clinically meaningful because the average distance after CR exceeded the threshold of 300 m [22].

The impact of exercise on mortality, hospital admissions, aerobic capacity, and QOL for both categories of HF have varied among previous studies. In the HF-Action study on patients with HFrEF, after statistical adjustment, exercise was associated with a modest but significant reduction for both all-cause mortality and hospitalization, including cardiovascular mortality and heart failure hospitalization, as well as significant improvements in 6MWT and VO2peak [19]. A meta-analysis by Chan et al. on patients with HFpEF found that exercise training led to significant improvements in VO2peak, HRmax, 6MWT, diastolic function, and QOL [3]. Borlaug et al. also observed improved aerobic capacity and QOL, and a 50% reduction in HF hospitalizations following exercise interventions for patients with HFpEF [1]. A recent study on patients with HFpEF by Mueller et al. found a significant improvement in VO2peak for both high-intensity interval training and moderate-intensity training compared to a no-exercise control group at 6 months but no significant differences between the three groups at 12 months [23]. A 2023 scientific statement by the AHA and ACC declared that exercise capacity improvements were similar or greater in those with HFpEF compared to those with HFrEF [2]. This is supported both by our findings and those of Pandey et al., who studied patients with HFrEF and HFpEF and found greater improvements in VO2peak in the patients with HFpEF following CR [6].

Regarding the impact of exercise on QOL and depression, our study found significant improvements in PHQ9 and F&P QOL following CR for both patients with HFrEF and HFpEF, which is supported by the previous literature on this topic [18,21]. Nolte et al. found that patients with HFpEF had a significant improvement in PHQ-9 scores following exercise, whereas the control group did not [18], and Davidson et al. found a significant improvement in MLHFQ QOL scores following CR in patients with both HFrEF and HFpEF [21]. Currently, there are fewer treatment options for patients with HFpEF compared to those with HFrEF, yet exercise seems to be a promising adjunct therapy option to address these functional limitations and impairments [2].

### 4.3. Importance of Cardiopulmonary Rehabilitation

Our study is unique as we looked at the impact of CR, not just exercise training. While the primary symptom of HF is exercise intolerance, patients often have comorbidities that also impact their physical and emotional state. While both HFrEF and HFpEF share most of the traditional HF risk factors, the risk for HFpEF appears to be associated with obesity, insulin resistance, and physical inactivity more so than HFrEF [1]. Therefore, aerobic exercise alone does not offer the best intervention to address these co-morbid conditions, along with the balance impairments and peripheral and respiratory muscle weakness that are often present [24,25,26]. Thus, supervised exercise combined with education, risk factor modification, and behavioral and psychological interventions by a multidisciplinary team is warranted [2]. Our study appears to be the first to include such a comprehensive physical and emotional assessment of patients with both HFrEF and HFpEF in the setting of CR.

Similarly to our study, Pandey et al. assessed patients with both HFrEF and HFpEF and found no significant difference in baseline aerobic capacity but significant improvements in both groups following CR, with HFpEF having greater VO2peak improvements [6]. Although no significant differences in aerobic capacity were observed at baseline in the Pandey study, our study found that patients with HFpEF performed significantly worse on the initial 6MWT, reflecting poorer aerobic/functional capacity. Our intervention results are consistent with Pandey et al.’s findings of significant improvements in both HF groups and greater improvement in VO2peak for patients with HFpEF [6].

Our study adds to the growing body of evidence, indicating that patients with HFpEF have risk factors, physical limitations, and QOL levels that are comparable to patients with HFrEF and that they benefit from supervised exercise, including CR, to a similar or greater magnitude than their HFrEF counterparts. Current guidelines recommend CR as Class 1 (Level of Evidence A) for exercise training in patients with HF, even though this recommendation does not distinguish between HFpEF and HFrEF and is largely based on patients with HFrEF [2]. Our findings, along with the growing understanding of HFpEF’s pathophysiology and limited pharmacological management, highlight the need for insurance coverage for CR, through which patients can receive supervised exercise training and comprehensive multidisciplinary care for risk factors, including education and referrals to other healthcare providers.

This study has several strengths. First, we included both patients with HFrEF and HFpEF using the most updated definitions. Our group included older patients and women, both of whom are under-researched groups, and included a large number of subjects with nearly equal numbers in each group. In addition, all patients attended the same CR program over the 5 years with the same equipment, facility, and staffing. Furthermore, the outcome measures used cover a variety of clinical aspects, from anthropomorphic measures to functional performance and from strength and balance to depression risk and QOL.

The limitations of this study include its retrospective nature and lack of a control group, as well as its reliance on sometimes incomplete medical records. This study included only one facility, decreasing its generalizability, yet we believe this to also be a strength due to consistent staffing and components of the individualized plan of care. Each patient received an individualized treatment plan based on their initial evaluation and goals, which is appropriate for a CR program, aiming to achieve a moderate to high intensity based on a variety of physiologic parameters. Therefore, the exercise programs were not identical among patients, which may decrease the study’s generalizability and reproducibility. Although we did not collect data on race or ethnicity, the CR was provided in Miami, Florida, and included a substantial number of Hispanic patients, which may not be reflective of the general population. However, this is potentially a strength of our study, as this group is also under-represented in CR. While the number of subjects in our study is smaller than other papers on HF, it is consistent with or greater than other studies including an exercise intervention and/or CR in patients with HF that include a significant proportion of patients with HFpEF. Lastly, our program staff included a variety of healthcare professionals, including physical therapists, which may not reflect typical staffing in a CR program and thus decreases the findings’ generalizability to all CR programs.

## 5. Conclusions

In conclusion, patients with HFpEF have similar, and sometimes greater, impairments compared to patients with HFrEF, and both HF subtypes have similar statistically and clinically significant improvements following CR. These results support the AHA and ACC’s 2023 scientific statement [2] advocating for insurance coverage for supervised exercise training, such as CR, for patients with HFpEF.

## Figures and Tables

**Table 1:** Baseline characteristics of study participants.

	HF with Preserved EF (>50%), *n* = 56	HF with Reduced EF (<40%), *n* = 43	*p* Value
Demographics			
Age, yr	68.9 ± 11.7	62.2 ± 13.7	0.01
Female	19 (34%)	12 (28%)	0.66
Height, centimeters	169.2 ± 11.2	169.9 ± 13.0	0.8
Weight, kilograms	84.6 ± 20.0	83.7 ± 21.5	0.96
Body mass index, kg/m^2^	30.6 ± 5.1	28.4 ± 7.3	0.74
Number of sessions completed	33.7 ± 10.0	32.3 ± 6.1	0.45
Cardiovascular Measures, Risk Factors, Comorbidities, Medications			
Ejection fraction, %	58.6 ± 5.7	27.5 ± 7.8	0.001
Waist circumference, centimeters	103.9 ± 15.2	103.1 ± 19.3	0.74
Number of comorbidities	4.5 ± 2.8	4.4 ± 2.3	0.78
Number of medications	12.7 ± 5.9	11.0 ± 5.4	0.18
Functional and Emotional Outcome Measures			
6-minute walk test (6MWT), meters	299.6 ± 120.5	350.6 ± 95.4	<0.001
5-time sit-to-stand (5xStS), seconds	16.9 ± 6.5	15.1 ± 4.2	0.54
Timed-up-and-go (TUG), seconds	11.1 ± 3.6	10.2 ± 3.6	0.46
Patient Health Questionnaire (PHQ9)	4.8 ± 4.6	4.1 ± 4.5	0.56
Ferrans and Powers Quality of Life (F&P QOL)	24.0 ± 4.0	25.0 ± 3.7	0.42
BERG balance (BBS), 0–56 points	48.8 ± 8.8	51.5 ± 6.5	0.08

Data are presented as mean ± standard deviation or *n* (%). Abbreviations: EF, ejection fraction; HF, heart failure.

**Table 2:** Changes in outcome measures following cardiopulmonary rehabilitation.

	HF with Preserved EF Pre, *n* = 56	HF with Preserved EF Post, *n* = 56	*p* Value Within Group	HF with Reduced EF Pre, *n*= 43	HF with Reduced EF Post, *n*= 43	*p* Value Within Group	*p* Value Between Groups
6MWT, meters	299.6 ± 120.5	400 ± 124.6	<0.001	350.6 ± 95.4	450.2 ± 129.0	<0.001	0.07
5xStS, seconds	16.9 ± 6.5	13.0 ± 4.4	<0.001	15.1 ± 4.2	11.1 ± 3.8	<0.001	0.07
TUG, seconds	11.1 ± 3.6	8.8 ± 3.6	<0.001	10.2 ± 3.6	7.7 ± 2.9	<0.001	0.18
PHQ9	4.8 ± 4.6	1.9 ± 2.5	<0.001	4.1 ± 4.5	2.6 ± 2.6	<0.001	0.19
F&P QOL	24.0 ± 4.0	26.0 ± 4.2	<0.001	25.0 ± 3.7	27.2 ± 2.4	<0.001	0.18
BBS, 0–56	48.8 ± 8.9	51.9 ± 7.7	<0.001	51.5 ± 6.5	53.4 ± 3.6	<0.001	0.27
Weight, kilograms	84.6 ± 20.0	83.3 ± 81.5	0.1	83.7 ± 21.5	82.8 ± 20.2	0.26	0.62
Body mass index	30.6 ± 5.1	30.1 ± 4.8	0.05	28.4 ± 7.3	28.5 ± 7.6	0.74	0.44
Waist circumference, centimerers	103.9 ± 15.2	101.6 ± 13.5	<0.001	103.1 ± 19.3	101.6 ± 18.5	0.05	0.87

Data are presented as mean ± standard deviation or *n* (%). Abbreviations: 5xStS, 5-time-sit-to-stand; 6MWT, 6-minute walk test; BBS, BERG balance scale; EF, ejection fraction; F&P QOL, Ferran and Powers’ Quality of Life; HF, heart failure; PHQ9, Patient Health Questionnaire; TUG, timed-up-and-go.

## Data Availability

The raw data supporting the conclusions of this article will be made available by the authors on request.

## Abbreviations

The following abbreviations are used in this manuscript:

5xStS	5-time-sit-to-stand
6MWT	6-minute walk test
ACC	American college of cardiology
AHA	American heart association
BBS	BERG balance scale
BMI	body mass index
CR	cardiopulmonary rehabilitation
EF	ejection fraction
F&P QOL	Ferran and Powers’ Quality of Life
HF	heart failure
HFpEF	heart failure with preserved ejection fraction
HFrEF	heart failure with reduced ejection fraction
MLHFQ	Minnesota living with heart failure questionnaire
PHQ9	Patient Health Questionnaire
SD	standard deviation
TUG	timed-up-and-go
QOL	quality of life
